# Supplementary material for: The causal relationship between the human gut microbiota and pyogenic arthritis: a Mendelian randomization study
Source: Front Cell Infect Microbiol. 2024 Nov 26;14:1452480. doi: 10.3389/fcimb.2024.1452480 (PMC11629706; doi:10.3389/fcimb.2024.1452480)
Supplement: Supplementary file 1 [file DataSheet1.zip › mendelian test/ebi-a-GCST90027759.csv_forest.pdf]

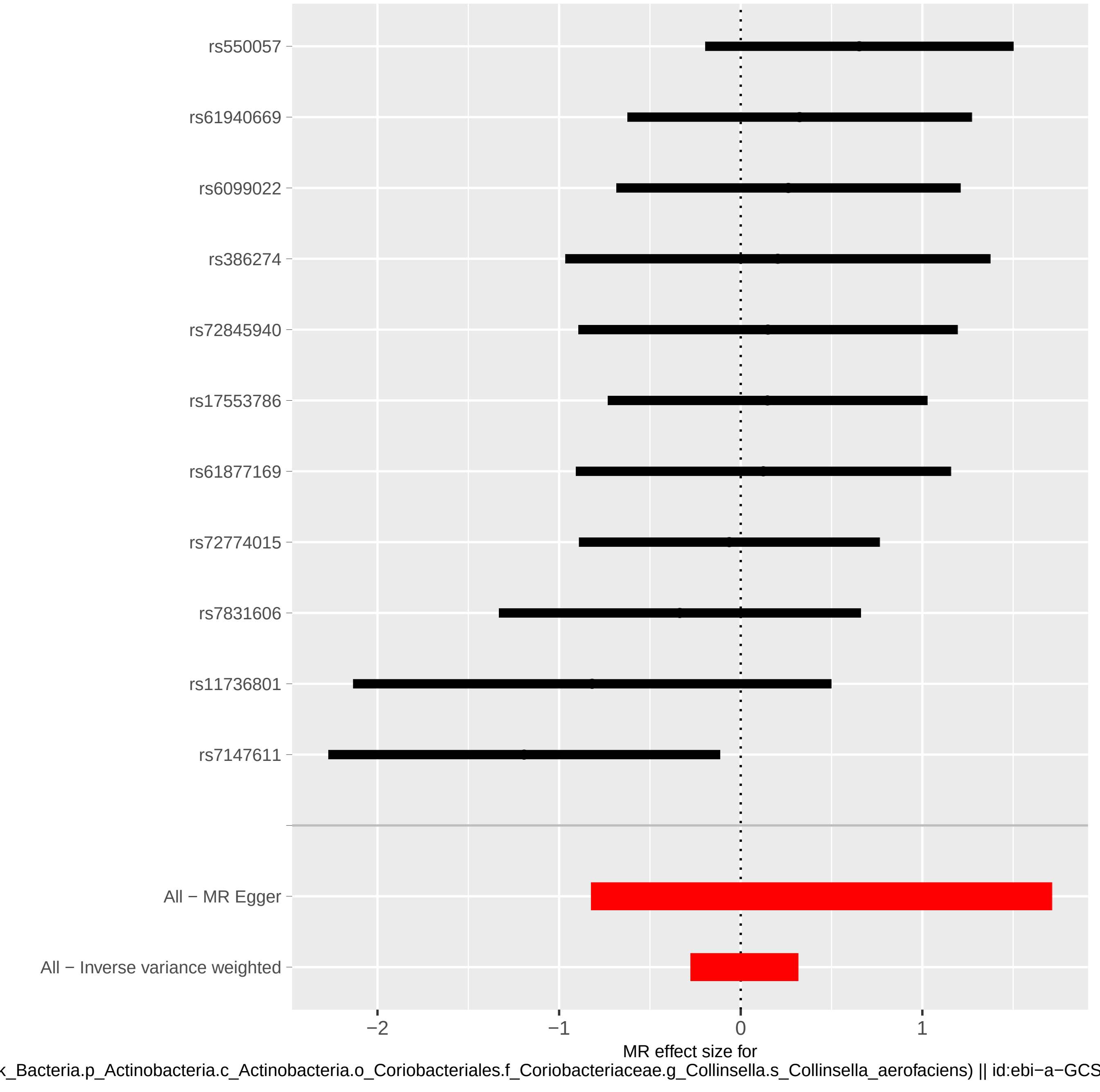

rs550057
rs61940669
rs6099022
rs386274
rs72845940
rs17553786
rs61877169
rs72774015
rs7831606
rs11736801
rs7147611
All − MR Egger
All − Inverse variance weighted
−2
−1
0
1
MR effect size for
k_Bacteria.p_Actinobacteria.c_Actinobacteria.o_Coriobacteriales.f_Coriobacteriaceae.g_Collinsella.s_Collinsella_aerofaciens) || id:ebi−a−GCS
